# Collective behaviors of Drosophila-derived retinal progenitors in controlled microenvironments

**DOI:** 10.1371/journal.pone.0226250

**Published:** 2019-12-13

**Authors:** Caroline D. Pena, Stephanie Zhang, Miles Markey, Tadmiri Venkatesh, Maribel Vazquez

**Affiliations:** 1 Department of Biomedical Engineering, City College of New York, New York, New York, United States of America; 2 Department of Biomedical Engineering, The State University of New York, Binghamton, NY, United States of America; 3 Department of Biomedical Engineering, Rutgers University, Piscataway, New Jersey, United States of America; 4 Department of Biology, City College of New York, New York, New York, United States of America; Massachusetts Institute of Technology, UNITED STATES

## Abstract

Collective behaviors of retinal progenitor cells (RPCs) are critical to the development of neural networks needed for vision. Signaling cues and pathways governing retinal cell fate, migration, and functional organization are remarkably conserved across species, and have been well-studied using Drosophila melanogaster. However, the collective migration of heterogeneous groups of RPCs in response to dynamic signaling fields of development remains incompletely understood. This is in large part because the genetic advances of seminal invertebrate models have been poorly complemented by in vitro cell study of its visual development. Tunable microfluidic assays able to replicate the miniature cellular microenvironments of the developing visual system provide newfound opportunities to probe and expand our knowledge of collective chemotactic responses essential to visual development. Our project used a controlled, microfluidic assay to produce dynamic signaling fields of Fibroblast Growth Factor (FGF) that stimulated the chemotactic migration of primary RPCs extracted from Drosophila. Results illustrated collective RPC chemotaxis dependent on average size of clustered cells, in contrast to the non-directional movement of individually-motile RPCs. Quantitative study of these diverse collective responses will advance our understanding of retina developmental processes, and aid study/treatment of inherited eye disease. Lastly, our unique coupling of defined invertebrate models with tunable microfluidic assays provides advantages for future quantitative and mechanistic study of varied RPC migratory responses.

## Introduction

The collective migration of retinal progenitor cells (RPCs) is fundamental to development, where heterogeneous RPCs of neuronal and glial lineages assemble the signaling networks critical for vision [1,2]. Collective cell movements differ significantly from the motion of individual cells, as cell clusters achieve locomotion via coordinated cell-cell adhesions [3–5] while singleton cells migrate largely independent of its proximal neighbors [6]. Few microfluidic systems have been adapted to study the collective behaviors of homogenous or heterogeneous cell groups [7–10] despite their wide usage in the chemotactic study of individual cells [7–11]. Microfluidic assays can significantly advance vision research by enabling quantitative study of the complex and poorly understood relationships between exogenous chemotactic fields and the collective RPC motility stimulated during retinogenesis [12–14].

**Fig 1:**
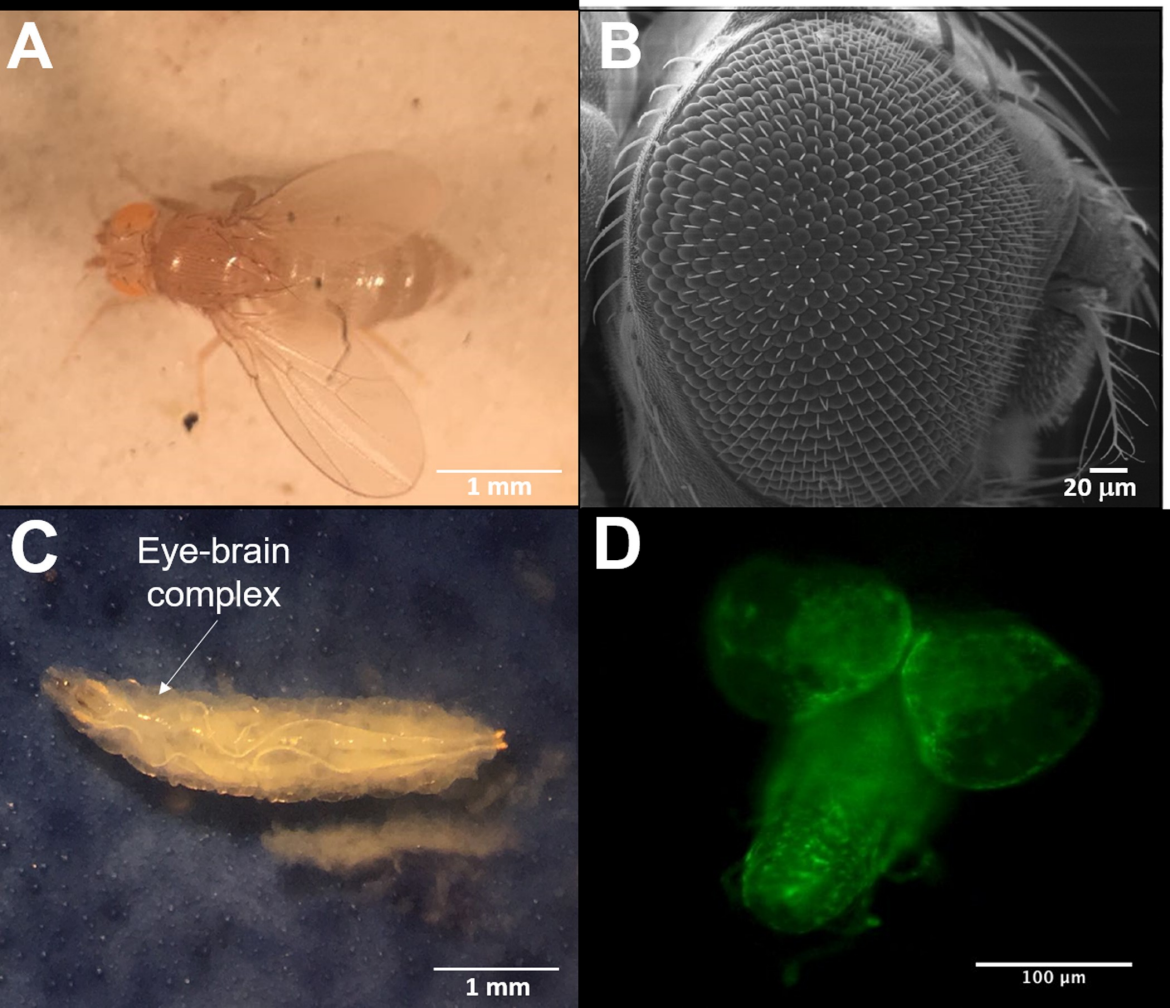
The developing visual system of a *Drosophila Melanogaster* invertebrate model. (A) Image of an adult fruit fly and (B) its compound eye examined via scanning electron microscopy (SEM). (C) Image of a Drosophila in the third instar stage of development, a post-embryonic, larval stage where retinal differentiation occurs. (D) A dissected eye-brain complex containing innate, heterogeneous populations of retinal progenitor cells (RPCs). Cells of glial lineage in this specimen are highlighted by GFP. Scale bars as shown.

Signaling cues governing cell migration in the developing visual system have been exceptionally well-studied using the invertebrate system of *Drosophila melanogaster*, or fruit fly [15–18]. Pathways ushering development of the ‘fly eye’ have been central to our evolving understanding of collective behaviors needed for retinal development across species [19–21]. The compound eye of an adult fly, shown in Fig 1, is comprised of approximately 800 ommatidia, or optical units, that communicate with visual centers in the brain [15,18,22]. Development of the compound eye requires the collective migration of heterogeneous RPC groups, i.e. both neuronal and glial progenitors, involving signaling pathways and mechanistic processes surprisingly analogous to vertebrate retinogenesis [19,23,24]. The combination of conserved pathways with significant genetic tools available, underscores Drosophila as a uniquely advantageous model with which to examine collective chemotactic responses of RPCs.

The current project isolated RPCs from the developing eye-brain complexes of Drosophila and examined their collective migratory responses to signaling gradients of fibroblast growth factor, FGF, a potent chemoattractant in its visual system [25,26]. We adapted a microfluidic assay to create time-dependent distributions of FGF concentration that represent the dynamic and non-linear signaling profiles of retinogenesis [4,13]. RPC migratory responses to signaling within the assay were seen to depend upon the average size of innately clustered cell groups. RPCs collections of 5–15 cells, i.e. small clusters, migrated longer distances in response to larger signaling gradients and with higher directionality. By contrast, large clusters of more than 15 cells traveled the largest distances in response to moderate gradient fields. Larger gradient fields yielded the shortest migration distances from large clusters and their lowest directionality of movement. RPCs migrating as individual cells illustrated non-directional movement in all signaling fields. These results point to significant but underexplored differences in the collective chemotactic responses of RPCs based on size. Quantitative study of these diverse collective responses will advance our understanding of developmental processes during retinogenesis, and aid study/treatment of inherited eye disease. Lastly, our unique coupling of defined invertebrate models with tunable microfluidic assays provides advantages for future quantitative and mechanistic study of varied RPC migratory responses.

## Materials and methods

### *Drosophila Melanogaster* fly stocks

The GAL4-UAS system [27] was used to produce flies whose neuronal and glial retinal progenitors (RPCs) expressed either red (RFP) or green (GFP) fluorescent protein, respectively. *Drosophila Melanogaster* stocks of UAS-8D12-RFP; Repo and UAS-mCD8-GFP; elav GAL4 were maintained on standard corn meal agar medium and kept at 25°C. Stocks were flipped or transferred once a week to maintain lines. Third instar larvae were removed from fly stock and dissected to extract their developing eye-brain complexes, as shown in Fig 1. Fluorescently-labeled RPCs (both GFP^+^ and RFP^+^) were then disassociated from eye-brain complexes for in vitro study.

### Isolation and culture of retinal progenitor cells (RPCs)

Eye-brain complexes of third instar larvae were dissected and dissociated using conventional protocols [28–30] performed in a laminar flow hood to promote sterility. Larvae were placed in 70% Ethanol (VWR, Randor, PA) and washed three times in autoclaved de-ionized (DI) water. Eye-brain complexes were dissected using stainless steel #5 tweezers in phosphate buffered saline (PBS) and washed once in Schneider’s medium (Thermo Fisher Scientific, Waltham, MA) supplemented in 10% (vol/vol) heat inactivated fetal bovine serum (FBS) and 1% (vol/vol) penicillin streptomycin (Gibco, Grand Island, NY) to remove excess cells and tissue. Eye-brain complexes were kept in 40 μL of PBS on ice to prevent degradation of tissue and cell death until 15–20 complexes were gathered. Complexes were incubated in a 1-mL volume of 0.5 mg/mL concentration of collagenase (Gibco, Grand Island, NY) at 25°C for 1 hr. Digested brain tissue was centrifuged at 2000 RPM for 5 minutes and then washed twice by re-suspending in 1 mL of supplemented Schneider’s medium. Tissue was mechanically disassociated into cell suspension via manual pipetting in 150 μL of supplemented Schneider’s medium (10 μL per brain) using a cell strainer to separate disassociated cells. Resultant cell solutions were inserted into glass petri dishes (uncoated glass control) and placed within in a Barnstead Labline L-C incubator at 25°C, the established cell temperature of this invertebrate system [29,30]. An immortalized S2 Drosophila cell line used as a control for the incubated environment [31]. The innate clustering of freshly-disassociated cells into heterogeneous RPC groups of different average sizes was left undisturbed for up to 48 hours.

### Fixing and staining of retinal progenitor cells (RPCs)

Cell suspensions were centrifuged at 2000 RPM for 8 minutes and then plated atop conjugated glass substrates for 30 minutes to facilitate cell attachment. Substrate surfaces were treated a priori with 100 μg/mL Poly-L-lysine (PLL, Sigma-Aldrich, St. Louis, MO, USA), 15 μg/mL Concanavalin A (Con-A, eBioscience, Carlsbad, CA, USA) or 80 μg/mL Laminin (LM, Gibco, Grand Island, NY, USA), and heated for 1 min on a hot plate at 100°C before cell addition. Substrates of uncoated glass were used as controls. RPCs were fixed in 40 μL formalin (4% formaldehyde) (Sigma-Aldrich, St. Louis, MO) for 15 minutes and then washed 3X with PBST (0.1% Triton X-100) (Sigma-Aldrich, St. Louis, MO). Primary glia-specific antibodies 8D12 anti-Repo (Developmental Studies Hybridoma Bank, Iowa City, IA) and neuron-specific Rat-Elav-7E8A10 anti-elav (Developmental Studies Hybridoma Bank, Iowa City, IA) were diluted in PBST and incubated with fixed cells overnight at 4°C. Unbound antibody was removed by washing the slide 3X for 2 minutes, and 2X for 10 minutes with PBST. Secondary antibodies Alexa Fluor 488 goat anti-mouse IgG (Invitrogen, Carlsbad, CA), and Alexa Fluor 594 goat anti-rat IgG (Invitrogen, Carlsbad, CA) were diluted in PBST and added to the slide. The substrates were incubated for 2 hrs at room temperature (25°C), washed 3X for 2 minutes, followed by 3X for 10 minutes, and then mounted with ProLong Diamond Antifade Mountant (Invitrogen, Carlsbad, CA).

### Measurement of retinal progenitor viability

The fraction of viable RPCs was measured after 24 hrs and 48 hrs on each treated substrate against control using the Colorimetric Cell Viability Kit III XTT (Invitrogen, Carlsbad, CA, USA). Reductions in viability were assessed by comparing XTT absorbance with values obtained from assays of newly-dissected cells (N = 15–20 eye-brain complexes, isolated as described). All absorbance values were normalized against those from controls (uncoated glass) to produce data within a range from 0 (100% cell death) to 1 (100% cell survival).

### The μLane assay: Design and operation

**Fig 2:**
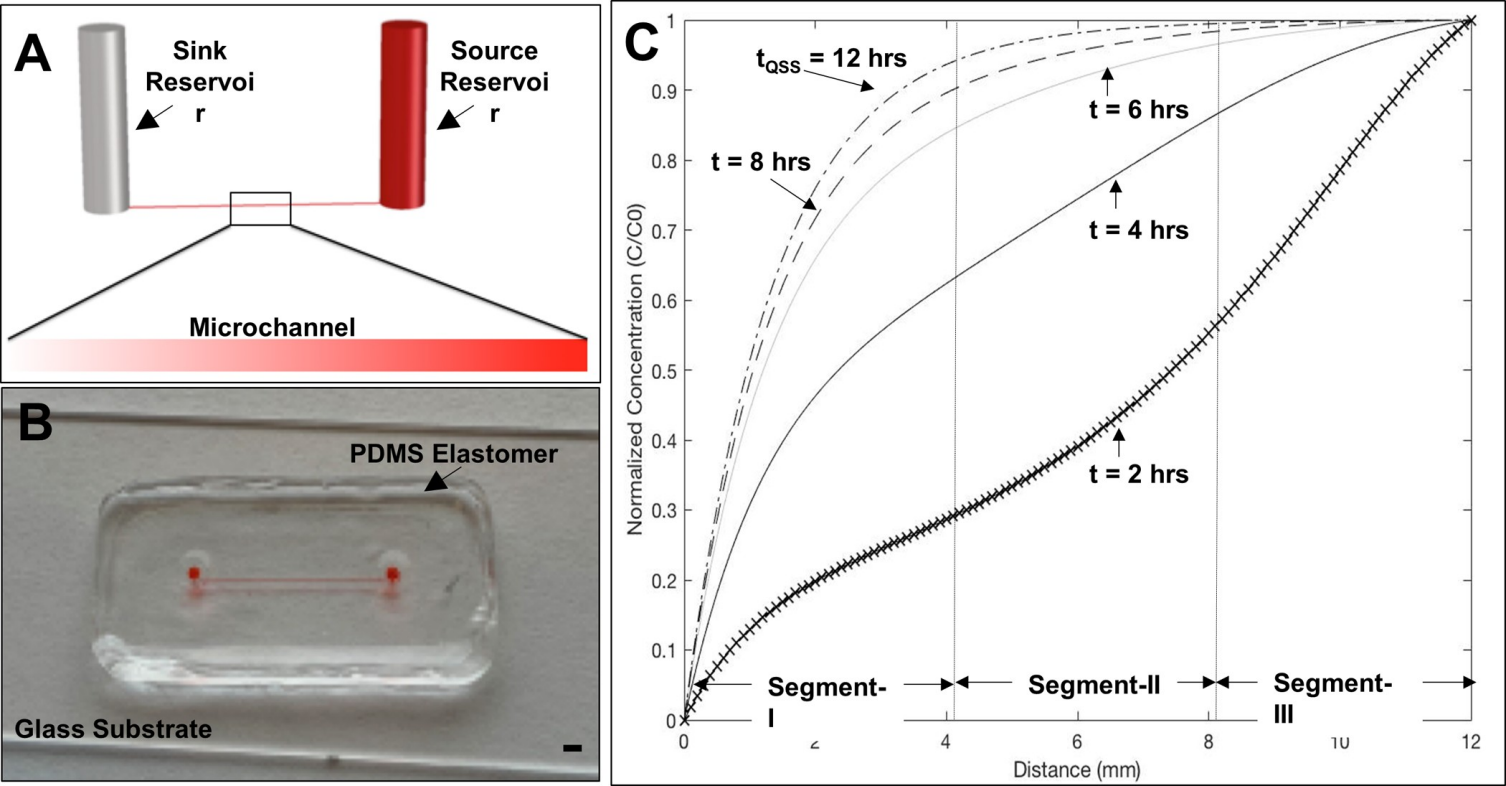
Description of the μLane assay and the non-linear signaling fields produced within its microenvironment over time. (A) Schematic of the microfluidic system comprised of two source and sink reservoirs connected by a 100-micron-diameter channel. Inset shows a representative concentration gradient field generated within the adjoining microchannel. (B) Image of PDMS fabricated device loaded with dye for visualization of its fluidic chambers. Scale = 1mm. (C) Distributions of FGF concentration, C(x,t), produced within the assay microchannel over time, normalized to the input concentration, C_o_. Sample distributions at t = 2, 4, 6, and 8 hrs are shown alongside t_QSS_ = 12 hrs. Segment-I, Segment-II, and Segment-III of the microchannel denote areas of mathematically-distinct changes in average FGF concentration, C, and gradient, G, over time.

The μLane system has been previously described by our group [32,33] and used to analyze chemotactic processes of cells derived from a variety of animal models. As shown in **Fig 2,** the current project used a μLane assay comprised of two large volume reservoirs, a source and a sink of 9-μL-volume each, connected by a microchannel of 100-μm-diameter and 12-mm-length. This geometry is micro-molded within a poly-dimethylsiloxane (PDMS) elastomer that is ozone-bonded to a chemically-cleaned (Nanostrip, VWR, MA) glass side or coverslip to create a closed microfluidic system. All inner surfaces of the assays were then treated with extracellular substrates of PLL, Con-A, and LM to facilitate migration study. Cells were seeded into the μLane cell reservoir, or sink, while FGF was added into its source reservoir. A time-dependent and transport-driven concentration gradient was then developed within the system microchannel, which stimulated RPC motility in response to changing signaling fields of FGF. RPC migration was recorded every hour within different spatial positions of the assay for a total of 8 hours, post cell seeding. Microdevices with respective reservoirs filled with cells and/or Schneider’s media (no FGF/gradients) were used as controls.

Transport within the microchannel was modeled using the well-established convective diffusion model [34–37], where the coupling of bulk flow with molecular diffusion creates non-linear concentration gradients described by Eq 1:

$$\frac{dC}{dt}+\bar{u}\cdot \bar{\nabla }C=D\cdot \nabla^{2}C$$ (1)

Where C denotes concentration in g/mL, t is time measured in s, u is bulk velocity in m/s and D represents molecular diffusivity in m^2^/s. Transport of FGF in the μLane assay established a quasi-steady-state concentration gradient, G, between the source and the sink reservoirs spanning several orders of magnitude, as shown in **Fig 2C**. A quasi-steady-state is defined here as a condition where the time to reach steady-state in the microchannel is much smaller than that required to change reagent concentration in the two adjoining reservoirs [38–40]. In this case, quasi-steady-state was reached after ~12 hours in the μLane (i.e. average changes along microchannel <5%) and maintained for an additional 3–4 days before reservoir concentrations begin to change measurably, i.e. by > 8–10%.

Concentration gradient fields of FGF (Invitrogen, Carlsbad, CA) within the assay were established by inserting a C_o_ = 100 ng/mL concentration of FGF into the source reservoir (reference point x_L_ = 1.2 cm) after the microchannel and sink reservoir were filled with RPCs suspended in media. The quasi-steady-state FGF distribution shown at t_QSS_ = 12 hrs was validated with experimental data and computational modeling within 2% error of one another. A bulk velocity of u = 0.37±0.06 μm/s was measured using 1.9-μm-diameter fluorescent beads (Duke Scientific, Palo Alto, CA, Cat. No. G0200) injected in the system and visualized via fluorescence microscopy over 24 hours, as done previously by our group [33,41–43]. A solution of Dextran (MW: 40kDa, Invitrogen, CA) was similarly inserted into the assay to validate formation of a quasi-steady-state gradient after ~ 12 hrs via measurements of fluorescent intensity, as also reported by our group. Additionally, the time-evolving solution to Eq 1 was modeled computationally via finite-element-analysis (FEM) in Matlab 7.7 (MathWorks, Natick, MA). The boundary conditions fixed the sink reservoir (x_0_ = 0 mm) at 0 ng/mL and the source reservoir (x_L_ = 12 mm) at 100 ng/mL. An initial condition of C(x, t = 0) = 0 ng/mL was set along the full microchannel length.

As seen in **Fig 2C**, the μLane generated highly non-linear concentration profiles that changed with time until reaching quasi-steady state. Distributions of FGF along the assay microchannel are shown at quasi-steady state (t_QSS_ = 12 hrs) as well as at select experimental times (t = 2, 3, 5, 6, 8 hrs) to illustrate the dynamic temporal and spatial changes in FGF signaling fields produced in the assay. As seen, FGF concentration profiles were non-linear at all time points studied, with different concentration gradients produced along different length segments of the μLane. The microchannel length was discretized into 100 equal segments per mm (as denoted by x marks on **Fig 2C**) to facilitate mean calculation of non-linear changes in concentration, C, and gradient, G, per mm of channel, x. For ease of analyses, spatial regions of the microchannel were divided into thirds, denoted as Segment-I, Segment-II, and Segment-III. These segments were chosen because mathematically distinct changes in FGF concentration gradient were produced along the segment lengths. Each of these gradients was approximately an order of magnitude apart from one another, for a range of 10^−1^ ≤ G ≤10^+1^ ng/mL per mm as summarized in **Table 1**. Lastly, because measurements of RPC movements represent a time-averaged response to changing distributions of gradient fields, the average time rate of change of FGF gradients, G_TRC_, we also calculated for each μLane segment. The non-linear G_TRC_ was mathematically computed using Eq 2:

$$G_{TRC}=\sum_{i=1}^{i=N}\left\{\frac{\partial }{\partial t}\left(\frac{\partial C\left(x,t_{i+1}\right)}{\partial x}\right)-\left(\frac{\partial C\left(x,t_{i}\right)}{\partial x}\right)\right\}$$ (2)

Where C is reagent concentration in ng/mL, x is channel length in mm, t is time, N is the number of time points studied (in this study N = 8), and ∂c/∂x is the concentration gradient, G, in units of ng/mL per mm of channel. RPC movement along different spatial coordinates of the μLane assay over time were related to the changes in the extracellular environment described in **Table 1**.

**Table 1:** Quantitative parameters used to describe the dynamic distribution of FGF molecules along the assay length. The spatial positions of Segment-I, Segment-II, and Segment-III are shown along microchannel length, x, measured in mm. Average values of the FGF gradient fields, G, in each segment are calculated in (ng/mL per mm of channel). The average range, R, and average percentage change in FGF concentration, μC, are shown in respective units of (ng/mL) and percent. The average time rate of change of gradient fields, G_TRC_, is shown in units of (ng/mL per mm) per hour.

	Assay Position (mm) x	Avg. Gradient Field (ng/mL per mm) G	Avg. FGF Conc. Range (ng/mL) R	Avg. % Change in Conc. ΔC	Avg. Time Rate of Change G_TRC_
**Segment-I**	0.0–4.0	^I^ G = 2.3 x 10^+1^	(78–16)	51%	0.04
**Segment-II**	4.1–8.0	^II^ G = 2.2 x 10^0^	(89–32)	55%	0.03
**Segment-III**	8.1–12.0	^III^ G = 4.4 x 10^−1^	(98–86)	22%	0.02

### Microscopy and imaging

#### SEM

An image of the adult *Drosophila* compound eye was produced via scanning electron microscopy. UAS-GFP adult Drosophila flies (N = 5) were coated with 20nm of gold utilizing the Cressington 308R Coating System (Cressington, Watford, England). Gold-coated flies were placed into the Zeiss LS704U Scanning Electron Microscope (Zeiss, Jena, Germany) and imaged at 6kV and 2.601A with the stage at a Z plane of 23.372 nm.

#### Confocal

A Nikon Eclipse TE2000 inverted microscope (Morell Instruments, NY) with a 20X objective was used in conjunction with the NIS Elements Imaging Software to gather fluorescent images of fixed and stained cells. Confocal images of fixed and stained cells were captured using a Zeiss LSM 800 (Zeiss, Jena, Germany) with Airyscan under 40X and 63X oil objective. An argon laser at 488 nm and 594 nm and was used to excite immunostained glial and neuronal progenitors, respectively.

#### Bright field

Images of cells adhered upon treated substrates were captured at 20X and 40X magnification using a Nikon Eclipse TE300. Bright field images of μLane devices were captured every 1 hr for 8 hrs along different segments of the assay.

### Parameters used for analysis

#### Numbers of RPCs

Total numbers of RPCs and average numbers of RPCs per eye-brain complex were calculated via optical microscopy using a hemocytometer and Trypan Blue. A total of N = 6 independent samples, per each of 3 dissection conditions, were examined to determine the mean numbers of individual and clustered RPCs.

#### Path length

Displacements of individual RPCs and RPC clusters were examined within Segment-I, Segment-II, and Segment-III of the μLane assay, and plotted over time using ImageJ with the Manual Tracking plugin (NIH, Bethesda, MD). All measurements of displacement were marked using the center of mass of single cells and of RPC clusters at each time point. The total path length, PL, or sum of the cell distances travelled from point to point was determined using Eq 3:

$$PL=\sum_{i=1}^{n}=\sqrt{\left|{\left(X_{i+1}-X_{i}\right)}^{2}+{\left(Y_{i+1}-Y_{i}\right)}^{2}\right|}$$ (3)

where X and Y represent spatial positions of individual RPCs and RPC clusters within the μLane at two consecutive time points. The path of RPC centers of mass was used to create cell trajectories, plotted using normalized X and Y spatial coordinates for the time points recorded. Conventional methods were used to perform time-lapsed cell studies, as per previous studies from our group and that of others [9,44–47]. Representative trajectories describe the average movement of individual cells (IC), small clusters (SC), and large clusters (LC) in each FGF signaling field.

#### Chemotactic index

The chemotactic index, CI, was calculated for individual RPCs (n = 1992), small clusters (n = 224), and large RPC clusters (n = 198) within the different segments of the μLane. Directional migration was determined using the CI, shown in Eq 4:

$$CI=\frac{ND}{PL}$$ (4)

where ND is the net cell displacement (μm) in the direction of the gradient field, and PL is the total path length (μm). Dimensionless values of CI approach 1 as cells move in the direction of increasing gradient and become negative when cells migrate away from gradient field. A value of CI ≥ 0.5 is used to denote directional migration, or positive chemotaxis, as conventionally defined by our group and others [9,10,48,49].

### Statistical tests

Statistical significance between experimental groups was evaluated using one-way Analysis of Variance (ANOVA) with 95% confidence interval and a post-hoc test (Tukey) for comparing multiple samples. Data analyzed was gathered using multiple measurements (1992 individual RPCs, 224 small clusters, and 198 large clusters) from multiple experiments (5≤n≤8) performed using 3–5 independent in vitro devices (glass substrates, microfluidic assays). ANOVA was used to determine statistical significance between control and experimental groups, while the post-hoc (Tukey) test was used to evaluate significance across experimental groups. Statistically significant values of p < 0.05 were denoted with a single asterisk (*), while significant values of p <0.01 were marked with a double asterisk (**). Error bars denote the full range of data in all cases.

## Results and discussion

### Collective behaviors of retinal progenitor cells during development

Contemporary knowledge of the visual system has been significantly advanced through genetic study of retinal development in Drosophila Melanogaster [2,15,22]. Extensive scrutiny of this seminal invertebrate model has illustrated that vision-critical processes are highly-conserved across species, and occur within retinal architecture that is developed through the precise, collective chemotaxis of its varied progenitor groups [12,13,50,51]. Clusters of RPCs, containing cells of both neuronal and glial lineage, rely upon complex cell-cell interactions to maintain the cohesiveness of their collective behavior [5,13,52,53]. However, while the fly eye provides a wealth of molecular and signaling data to describe retinogenesis [24,54], its genetic advances have been poorly complemented by controlled, cell study of its visual development, in vitro. As a result, the collective chemosensitivity of heterogeneous progenitor groups during retinogenesis remains incompletely understood. Tunable microfluidic assays able to replicate the miniature cellular microenvironments of the developing visual system provide newfound opportunities to probe and expand our knowledge of collective RPC migratory responses essential to visual development across species. Our project is among the first to examine collective behaviors of primary Drosophila RPCs in vitro [55,56], and correlate their collective responses with dynamic fields of diffusible signaling molecules. Experiments first evaluated in vitro RPC viability, total cell numbers, and innate RPC clustering per eye-brain complex. These results provide significant primary data whose absence from contemporary cell-based vision research has limited adaptation of primary cells from invertebrate models for in vitro study. Our study then examined the motility of innately clustered, heterogeneous RPC groups in response to defined spatial and temporal gradients of FGF signaling.

### Heterogeneous retinal progenitor cells per eye-brain complex

**Fig 3:**
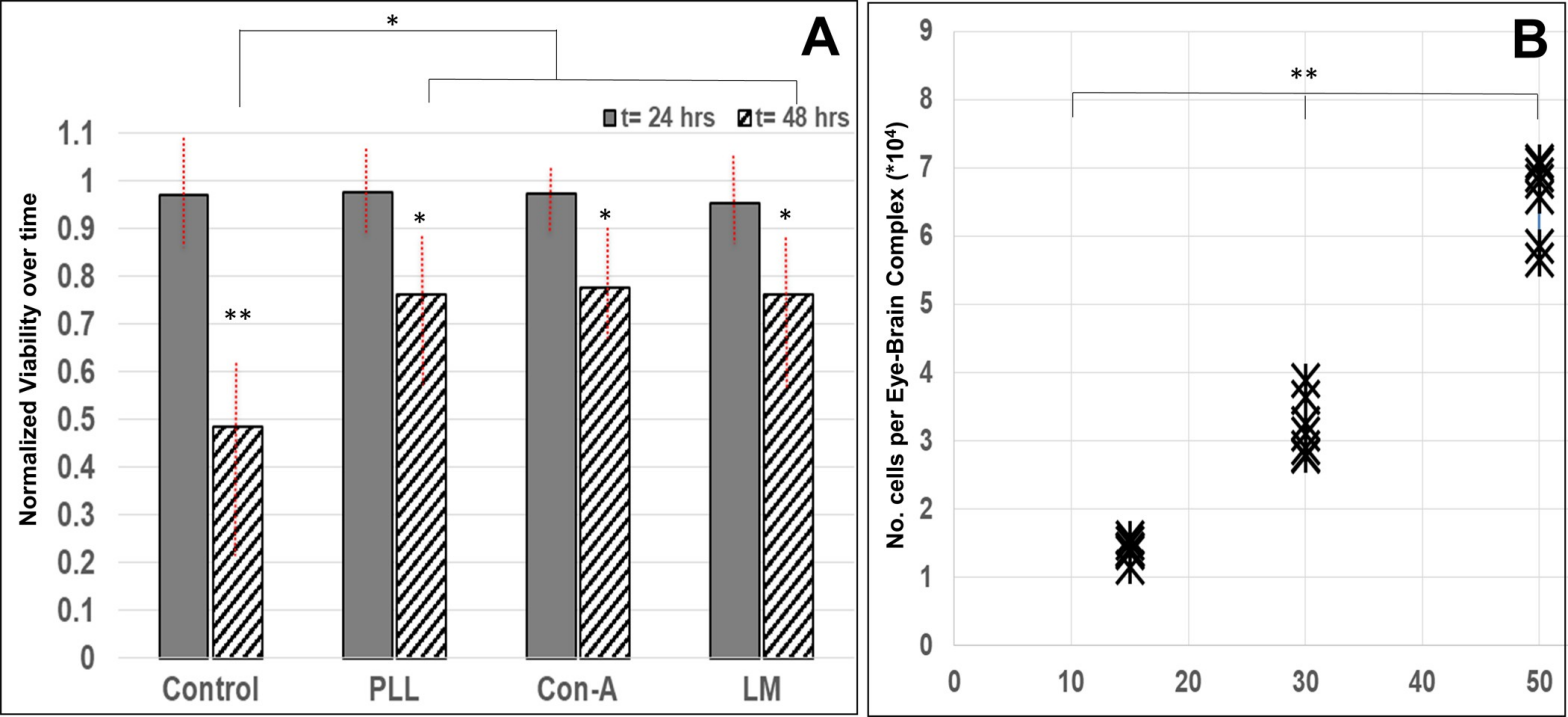
Total numbers of retinal progenitor cells (RPCs) disassociated from eye-brain complexes and their survival rates, post-dissection. **(A)** Measured changes in RPC viability upon substrates treated with extracellular substrates of poly-L-lysine (PLL), concanavalin A (Con-A), and laminin (LM) normalized against controls. Statistical differences were measured between each time step per substrate, but not across substrates. Statistical significance is denoted by ** (p<0.01) and * (p<0.05). (B) Average numbers of total cells per dissections of N = 15, 30, and 50 eye-brain complexes from third instar larvae. Data from 6 independent experiments, per dissection grouping, are denoted by an X. Statistical significance (**p < 0.01) was measured across all groups.

Few Drosophila projects have complemented genetic study with in vitro cell data, in part because of the difficulties experienced in sustaining its isolated progenitors via traditional culture [56]. However, wide biological adaptation of microfluidic devices has exploited the nano- to microliter volumes of these quantitative systems to produce suitable culture environments for a variety of primary cells, both for short and long term studies [9,11,57]. Reported viability of Drosophila cells as low as 12% over 24 hours [29,30,56] has greatly limited the in vitro applicability of its RPCs. Our project modified traditional dissections of eye-brain complexes with sterility protocols of mammalian cell culture and incorporated the use of different substrates (PLL, Con-A, and LM) to increase RPC survival. As shown in **Fig 3A**, solutions of isolated RPCs placed upon treated glass substrates exhibited levels of RPC viability similar to one another, and to controls (uncoated glass), after 24 hours, with 70–74% survival. This primary data was the impetus for performing in vitro measurements immediately post-dissection and for short, 8 hr times that maintained an 80–90% cell viability. As seen, RPC viability decreased by 50% in glass dishes after a total of 48 hours, but by a much lower 12% upon treated substrates (as measured by XTT). Statistical significance was measured between 24 and 48 hrs for each substrate, but not across substrates. At 48 hours, statistical significance was only recorded between viability of control and of the Con-A substrate.

Measurements of in vivo RPC cell density were also performed to best represent those ratios in vitro. RPC density in vitro is highly significant because the number and proximity of cell-cell interactions greatly influence its collective cell behaviors [58–61]. Here, we leveraged the unique wealth of Drosophila data for direct comparison of total cell numbers and cell lineage over different stages of retinal development. We note that cells isolated from developing eye brain complexes of the third instar stage are neuroblasts, known to only differentiate into retinal neurons or glia during the later stages of development [28,62]. These RPCs have been shown to respond to stimuli collectively, in vivo, by a variety of studies using genetics with live imaging techniques [28,63] as well as conventional fixation over time [4,55,64]. Average numbers of GFP^+^ (glial) and RFP^+^ (neuronal) RPCs were measured from 6 independent experiments, each, using N = 15, 30, and 50 eye-brain complexes, as shown in **Fig 3B**. Respective numbers of disassociated RPCs were N_15_ = 1.4x10^5^ cells for 15 eye-brain complexes, N_30_ = 2.9x10^5^ cells for 30 eye-brain complexes, and N_50_ = 6.8x10^5^ cells for 50 eye-brain complexes. Statistical significance (p < 0.01) was measured across all groups. These data produced an average value of N_RPC_ = 1.08x10^4^ RPCs per eye-brain complex, which is remarkably in line with the Drosophila literature. The adult fly eye is comprised of approximately 1.6x10^4^ cells in total, of which 1.1x10^4^ cells have neuronal and/or glial lineage, i.e. RPCs [17,18,20]. Our results thereby illustrate accuracy and reliability in isolating RPCs from Drosophila alongside large increases in cell survival. These contributions provide a significant step towards utilizing the developing fly eye, in vitro, to expand our understanding of collective behaviors during visual development.

### Clustering of primary retinal progenitor cells

Isolated RPCs were examined for the clustering behaviors innate to developing in vivo systems upon treated substrates and controls (untreated glass). Primary RPCs were observed to self-assemble and remain in clustered, heterogeneous groups 2–3 hours, post-dissection, for all cases. RPCs were seen to survive and adhere upon treated substrates as individual cells as well as within clustered groups. Numbers of clusters exceeded those of individual cells in all tests. Three groups of RPCs were observed per substrate condition: (a) Individual cells, IC, defined as cells with minimal to zero discrete points of contact with adjacent cells through extensions or processes [65]; (b) Small clusters, SC, denoted as groups of 5 to 15 cells in close proximal contact with surrounding cells ≥ 75% of its membrane surfaces; and (c) Large clusters, LC, comprised of more than 15 cells in close proximal contact, as above.

**Fig 4:**
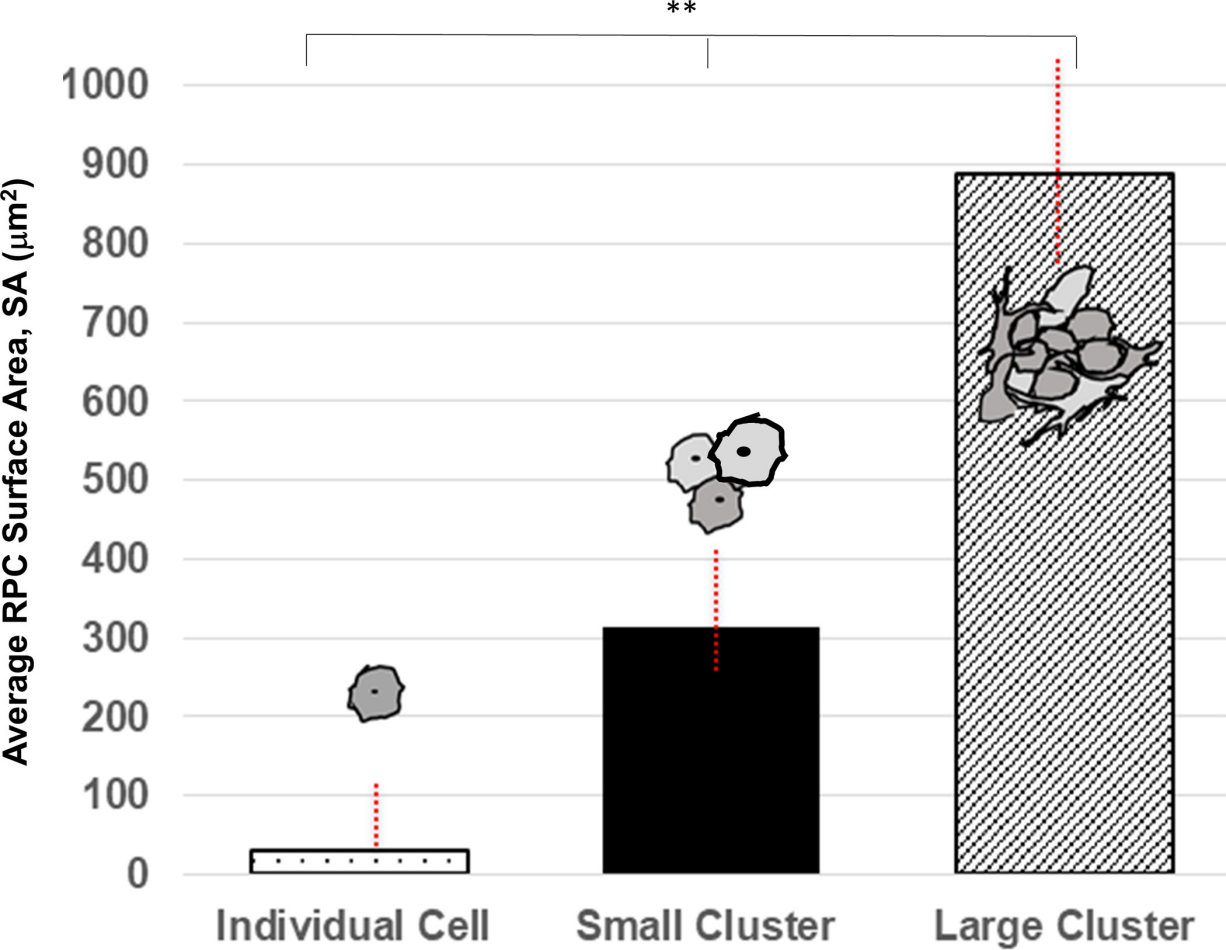
Mean surface area, SA, of retinal progenitor cells (RPCs) adhered as individual cells (IC), small clusters (SC), and large clusters (SC) of RPCs. An individual cell (IC) was defined as one without proximal cell-cell contract, as illustrated by the cell schematic. Small clusters (SC) of RPCs were denoted as groups of 5–15 cells in contact with neighbors on ≥ 75% of its cell membranes, as shown. Large clusters (LC) of RPCs were denoted by similarly interconnected groups of more than 15 cells, as per accompanying schematic. Statistical significance (** p < 0.01) was measured across all groups.

The average sizes of RPC clusters were estimated by measuring the adhered surface area, SA, upon substrate surfaces. However, differences in average RPC cluster size per treated substrate were not statistically-significant against one another (p>0.05: Data not shown). As such, the Con-A substrate was selected for all tests because of its applicability to visual systems across species and its wide usage in the Drosophila community [66–68]. As shown in **Fig 4**, the average surface area of individually-adhered cells (IC) was measured as SA_IC_ = 29.20 ± 10.65 μm^2^, while small clusters (SC) exhibited an average, adhered surface area of SA_SC_ 313.35 ± 167.51 μm^2^, and large clusters (LC) an average of SA_LC_ 873.73 ± 135.06 μm^2^. Statistical significance was measured across and between all groups (p<0.01), highlighting no overlap in the average size of each.

These data illustrate an innate preference for RPCs to remain in heterogeneous clusters of an optimal size range. This data underscores the significance of examining collective behaviors of RPC clusters of mixed neural lineage. While cell sorting can be used to generate homogeneous RPC groups, i.e. of only neuronal or glial cells, our data illustrate that innate heterogeneous clustering is most relevant to retinal study of the developing visual system. Further, we note that the small portion of RPCs able to survive and adhere as individuals was significant in each sample. As a result, motility tests will analyze their responses, albeit separately from RPC clusters. We note, however, that these individual cells migrate using well-studied mechanisms of cell crawling [8,45,69], while RPC clusters do not.

### Dynamic signaling fields of FGF within the microfluidic assay

Tests next utilized our μLane assay to produce signaling fields of FGF that varied with both spatial dimensions and elapsed time. The assay modelled the dynamic cellular microenvironments of the developing retina [26,70] by producing highly non-linear gradients over a testing period of 8 hrs. We note that an initial FGF concentration of C_0_ = 100 ng/mL was chosen based on the extensive study of its physiological relevance in Drosophila [26]. **Fig 2**illustrates the non-linear distributions of FGF signaling molecules, i.e. concentration, along the microchannel length, x, over time. As seen, the region denoted by Segment-I is located between x = 0 mm and x = 4.0 mm of microchannel, and produced an average change in FGF concentration of ^I^ΔC = 51% over the 8-hr duration of in vitro experiments. The average range, R, of FGF concentration was ^I^R = (78 ng/mL-16 ng/mL). These changes were highly non-linear and created an average FGF gradient field of ^I^G_I_ = 22.3 ng/mL per mm of channel, as per **Table 1**. We note that all distributions of FGF concentration per hour were discretized into 100 equal segments per mm of channel to facilitate more accurate estimates of average changes in non-linear concentration and gradient fields. This mathematical representation has been widely used for non-linear data with reported errors of less than 10% [71–73].

Segment-II of the assay is located mid-channel, between x = 4.0 mm and x = 8.0 mm, and produced an average change in FGF concentration of ^II^ΔC = 55%. However, this region produced average values of absolute FGF concentration that were much higher than Segment-I, with a range of ^II^R = (89 ng/mL- 32 ng/mL). These non-linear changes in concentration produced gradient fields an order of magnitude lower than the previous region, with an average gradient value of ^II^G = 2.2 ng/mL per mm of channel. Segment-III is located between x = 8.0 mm and x = 12 mm of the μLane assay, and produced the smallest average concentration change of ^III^ΔC = 22%. However, FGF concentration was highest in this region, with a range of ^III^R = (98 ng/mL—86 ng/mL). These values created very shallow gradient fields of signaling molecules, for an average gradient field of ^III^G = 0.44 ng/mL per mm of channel. Lastly, we note that cells in all segments of the μLane assay experienced gradients of signaling molecules that changed over time. As such, the average time rate of change of gradient fields within each segment, G_TRC_, was also calculated and shown in **Table 1**. However, these values were very similar to one another with ^I^ G_TRC_ = 0.04, ^II^ G_TRC_ = 0.03, and ^III^ G_TRC_ = 0.02 in respective segments, with units of ng/mL per mm of channel per hour.

### Migration of individual retinal progenitor cells (RPCs) towards FGF signaling

**Fig 5:**
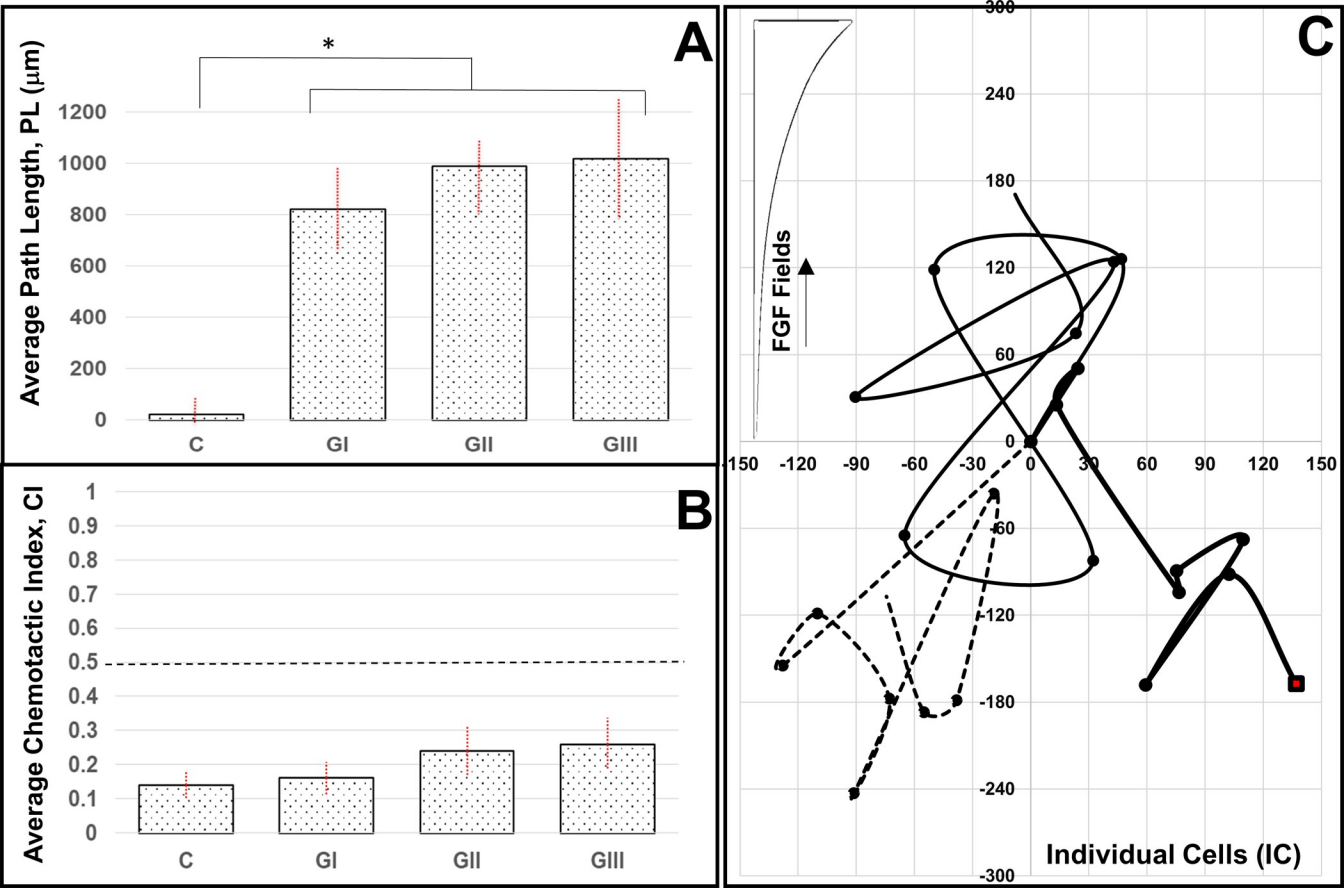
Migratory responses of individual retinal progenitor cells (RPCs) in response to FGF signaling fields produced in the μLane assay. **(A)** Average path lengths, PL, of small clusters (SC) and (**B**) mean values of chemotactic index, CI, in control conditions (No FGF/gradient) and in gradient fields ^I^G, ^II^G, and ^III^G generated within respective segments of the microfluidic assay. No statistical significance was measured against controls or across groups for PL (p > 0.05). A dashed line highlights CI = 0.5 to denote chemotactic migration. Statistical significance (** p < 0.01) is denoted between control and experimental groups of CI, but not across individual groups. (**C**) Representative cell paths, or trajectories, of individually motile RPCs within different FGF signaling fields of the μLane. Axes represent microchannel distances in microns (μm) and each RPC trajectory has been re-centered at the origin for ease of comparison. FGF signaling fields increase in the y-direction for all cell paths.

RPC migration in response to FGF signaling was evaluated using measurements of average path length, PL, and chemotactic index, CI, within the 3 segments of the microfluidic assay denoted. The average path length of motile, individual cells (IC) in response to FGF signaling fields from Segment-I was measured to be ^I^LP_IC_ = 819.4±79.1 μm, as shown in **Fig 5**. Average IC path lengths were ^II^PL_IC_ = 987.9±62.4 μm and ^III^PL_IC_ = 1018.6±119.8 μm in response to FGF signaling fields of Segment-II and Segment-III, respectively. Statistical significance (p < 0.01) was measured between control (no FGF/gradients) and all assay segments, but not across the changing gradient fields of each segment. In addition, the chemotactic index, CI, or directionality of IC movement, was measured to be very low, with values of ^I^CI_IC_ = 0.16 ± 0.21 in Segment-I, ^II^CI_IC_ = 0.24 ± 0.19 in Segment-II, and ^III^CI_IC_ = 0.26 ± 0.19 in Segment-III. No statistical significance was measured against controls or between groups (p>0.05). Values of CI less than 0.5 indicate non-directional movement and point to chemokinetic behavior stimulated by FGF concentration rather than concentration gradients that direct cell movement [42,47]. The chemokinetic response of IC was further observed in the representative RPC paths, or trajectories, of **Fig 5C**, which illustrate non-directional movement, both, along and against signaling gradients over time.

Together, these data illustrate non-directional migration of individually-motile RPCs in FGF signaling fields, and suggest that RPCs require cell-cell contacts and/or communication for directed movement in FGF signaling fields. Recent study has illustrated that RPC differentiation into retinal neurons and/or glia depends upon cell-cell adhesions that are also important for migration [53,74–76]. This correlation may suggest that individually-motile RPCs lack the ability to chemotax (i.e. directionally migrate) because they lack abilities to produce appropriate retinal architecture without neighboring cells [50,61,77,78]. Future study will take advantage of genetic manipulation of Drosophila to examine the influence of up/down regulation of cell-cell adhesion molecules on collective RPC chemotactic responses.

### Migration of clustered retinal progenitor cells (RPCs) towards FGF signaling

**Fig 6:**
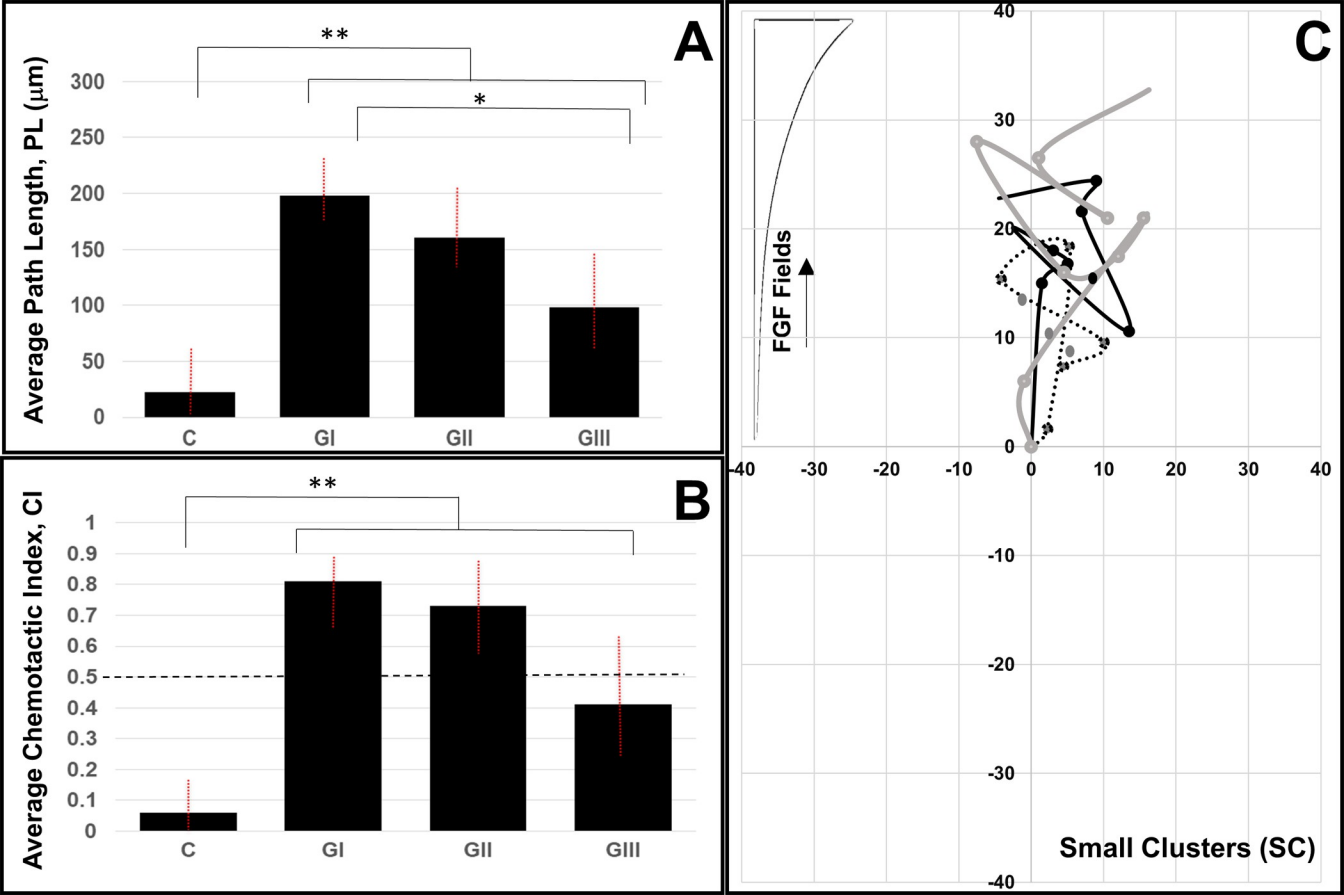
Migration of small clusters of retinal progenitor cells (RPCs) in response to FGF signaling fields produced in the μLane assay. **(A)** Average path lengths, PL, of small clusters (SC) and (B) mean values of chemotactic index, CI, in control conditions (No FGF/gradient) and in gradient fields ^I^G, ^II^G, and ^III^G generated within respective segments of the microfluidic assay. A dashed line highlights CI = 0.5 to denote chemotactic migration. Statistical significance (** p < 0.01) is shown between control and all experimental groups and across different combinations (* p < 0.05). (C) Representative SC paths, or trajectories of small clusters, in response to the gradient signaling fields in different segments of the assay. Axes represent distances in the microchannel (μm) and each RPC trajectory has been re-centered at the origin for ease of comparison. FGF signaling fields increase in the y-direction for all cell paths.

Final experiments examined the collective migration of RPC clusters in response to dynamic signaling gradients of FGF. As shown in **Fig 6**, small RPC clusters (SC), i.e. of 5–15 cohesive RPCs, exhibited path lengths of ^I^PL_SC_ = 97.8±21.8 μm, ^II^PL_SC_ = 161.5±10.2 μm, and ^III^PL_SC_ = 187.4±21.9 μm in Segment-I, Segment-II, and Segment-III of the μLane assay, respectfully. Note that these segments produced the same gradient fields, ^I^G, ^II^G, and ^III^G, as listed in **Table 1** and used for study of individual cells. Statistical differences (p < 0.01) were measured between control and each experimental group, but not across all groups. As seen, only path lengths at the highest, i.e. steepest, gradient, ^I^G, were significant against the PL measured in lower signaling fields (p < 0.5). Similarly, average CI values were measured as ^I^CI_SC_ = 0.81 ± 0.14, ^II^CI_SC_ = 0.72 ± 0.12, and ^III^CI_SC_ = 0.39 ± 0.14 in respective gradient fields. Representative cell paths, or trajectories, of small clusters illustrated net movement in the direction of increasing FGF gradients, as per **Fig 6C**. However, as values of CI ≥ 0.5 indicate directional migration, small clusters were shown to chemotax in response to the larger ^I^G and ^II^G fields, but not to the lowest, i.e. most shallow, gradient field of ^III^G.

**Fig 7:**
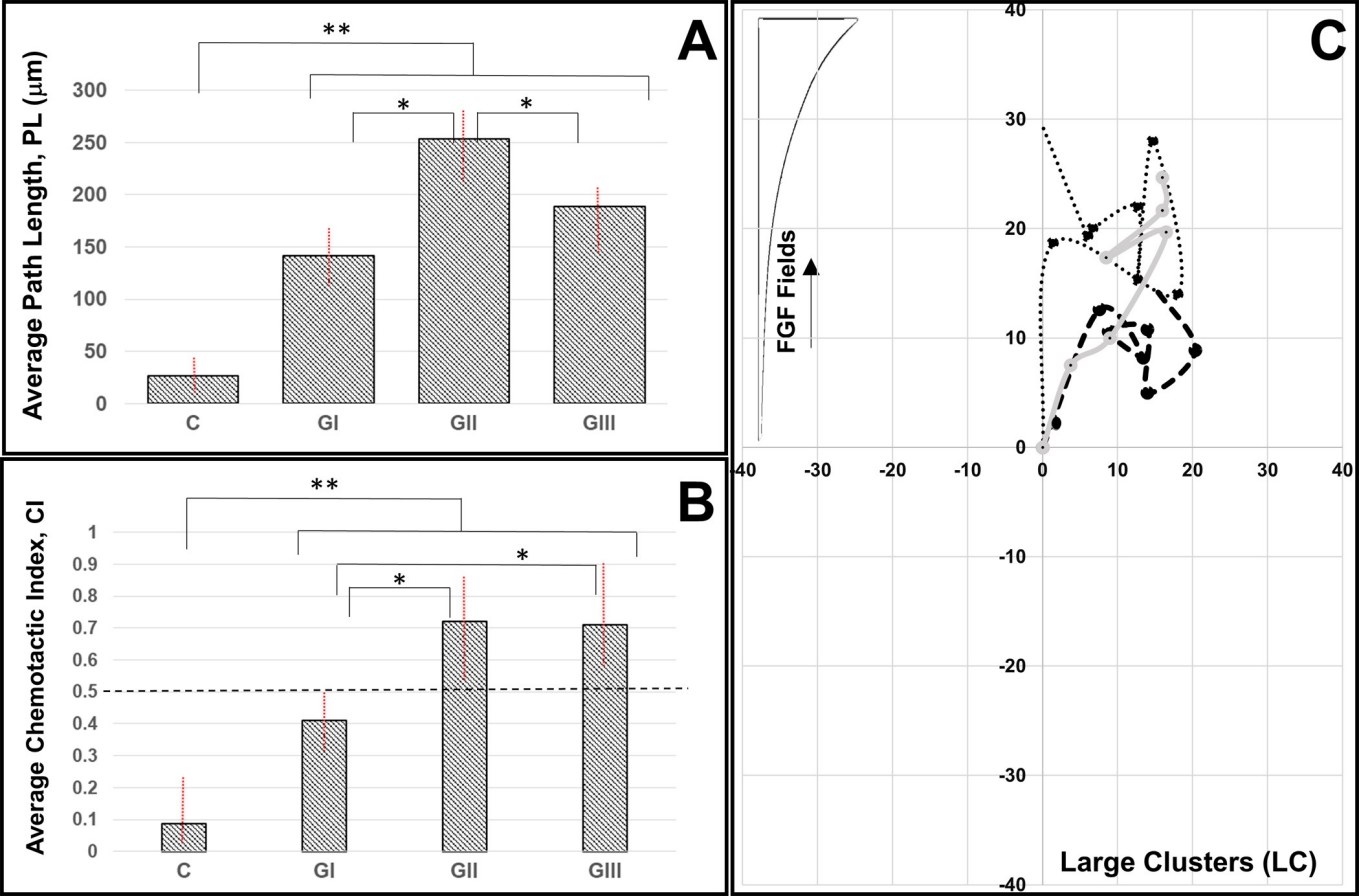
Migration of large clusters (LC) of retinal progenitor cells (RPCs) in response to FGF signaling fields produced in the μLane assay. **(A)** Average path lengths, PL, of large clusters and (**B**) mean values of chemotactic index, CI, in control conditions (no FGF/gradients) and in gradient fields ^I^G, ^II^G, and ^III^G generated within respective segments of the microfluidic assay. Statistical significance (** p < 0.01) is shown between control and all experimental groups, and across different combinations (* p < 0.05). (**C**) Representative cell LC paths, or trajectories of large clusters, in response to the gradient signaling fields in different segments of the assay. Axes represent distances in the microchannel (μm) and each RPC trajectory has been re-centered at the origin for ease of comparison. FGF signaling fields increase in the y-direction for all cell paths.

RPCs in large clusters (LC: comprised of 15 or more cells) illustrated similar average path lengths of ^I^L_LC_ = 141.3±23.6 μm in FGF signaling fields of Segment-I, ^II^L_LC_ = 253.6±32.1 μm in Segment-II, and ^III^L_LC_ = 188.9±31.3 μm in fields of Segment-III. Statistical significance (p < 0.01) was measured between control and each gradient group, but not across groups. As seen in **Fig 7A**, path lengths were only statistically different against fields of Segment II (p < 0.05). Representative LC paths, or trajectories of large clusters, illustrated net movement in the direction of increasing FGF gradients, with average CI values of ^I^CI_LC_ = 0.41 ± 0.16, ^II^CI_LC_ = 0.72 ± 0.20, and ^III^CI_LC_ = 0.71 ± 0.10 in respective gradient fields. However, no statistical significance was measured between ^II^G and ^III^G fields (p > 0.05). These data illustrate that large clusters do not migrate directionally in the largest gradient fields of Segment-I, but do chemotax in the moderate gradient fields of Segment-II and shallow gradients of Segment-III.

Taken together, the results of motile clusters suggest that collective chemotactic movement of RPCs is a function of average size. SC were able to respond to increasing gradients with increasing directionality and path lengths, as typical of conventional chemotactic behavior, while large clusters exhibited longer and more directional migration in response to signaling from moderate gradient fields. These differences may be a function of the number of cell-cell contacts between larger groups of RPCs. The inner cells of large clusters are the most surrounded by adjacent RPCs, indicating a higher number of cell-cell adhesions per RPC than the outer cells most directly exposed to biochemical stimuli. In conventional leader-follower models of collective migration [3,5,14], polarization is achieved by cells closest to the gradient stimulus, i.e. outer cells, which in turn initiate mechanical forces than drag adjacent cells along the chemotactic path, or trajectory. Such mechanical transmission through cell-cell adhesions plays a key role in the directed migration of RPC clusters that can either aid or retard collective chemotaxis. Recent studies have demonstrated that geometrically controlled cluster sizes produced active cell-cell contacts in smaller clusters that aided directionality [79]. Conversely, larger clusters exhibited more passive cell-cell adhesions that retained cluster cohesion during motion, but had little influence on its directionally. This phenomenon may be underlying the differences in our measurements of SC and LC migratory responses to FGF signaling.

In addition, we note that large clusters may have achieved displacement, in part, via rotation about its center of mass, rather than by direct displacement of its center of mass. Such motility has been particularly reported for multi-cellular systems, due to increasingly complex interactions between cell-cell adhesions and communication across cell types [80]. Future study will exploit the wealth of Drosophila genetic manipulation to examine the influence of up/down regulated cell-cell adhesion molecules in the chemotactic response of RPC clusters.

## Conclusion

Results of this project illustrate a size-dependent chemotactic migration of RPC clusters in response to FGF signaling. Unexpectedly, large RPC clusters illustrated chemosensitivity to more shallow gradient fields, while smaller clusters traveled larger directional distances with increasing gradient fields. These differences are likely due to the number and nature of cell-cell adhesions among heterogeneous RPC clusters of different size. The coupling of microfluidic assays with the exemplary genetic model of Drosophila will enable future mechanistic study of the complex relationships between cell-cell adhesion molecules and chemotactic receptors of clustered RPCs. Microfluidic systems better customized to the physiological dimensions/geometry of the developing eye will help elucidate properties of intrinsic RPC clustering and migration during different stages of retinal development across species. Lastly, we emphasize that while viability constraints remain significant to in vitro testing of primary RPC, these limitations can be greatly eased by microfluidic designs that achieve and/or maintain desired chemical environments as rapidly as possible.

## Supporting information

S1 Table Raw data files. Full set of computational solutions to transport within the microfluidic device and summary of experimental data.
(XLSX)
